# Autism Spectrum Disorder Symptom Profiles in Fragile X Syndrome, Angelman Syndrome, Tuberous Sclerosis Complex and Neurofibromatosis Type 1

**DOI:** 10.1007/s10803-024-06557-2

**Published:** 2024-10-12

**Authors:** Kyra Lubbers, Kamil R. Hiralal, Gwendolyn C. Dieleman, Doesjka A. Hagenaar, Bram Dierckx, Jeroen S. Legerstee, Pieter F.A. de Nijs, André B. Rietman, Rianne Oostenbrink, Karen G.C.B. Bindels-de Heus, Marie-Claire Y. de Wit, Manon H.J. Hillegers, Leontine W. ten Hoopen, Sabine E. Mous

**Affiliations:** 1 Erasmus MC Center of Expertise for Neurodevelopmental Disorders (ENCORE), Erasmus MC, Rotterdam, The Netherlands; 2 Department of Child and Adolescent Psychiatry and Psychology, Erasmus MC, Rotterdam, The Netherlands; 3 Child Brain Center, Erasmus MC, Rotterdam, The Netherlands; 4 Department of Pediatrics, Erasmus MC, Rotterdam, The Netherlands; 5 Department of Neurology, Erasmus MC, Rotterdam, The Netherlands; 6 Research Institute of Child Development and Education, University of Amsterdam, Amsterdam, The Netherlands; 7 Department of Child and Adolescent Psychiatry, Amsterdam University Medical Center/Levvel, Amsterdam, The Netherlands; 8 Full Member of the European Reference Network on Genetic Tumour Risk Syndromes (ERN GENTURIS)–Project ID No 739547, Amsterdam, The Netherlands

**Keywords:** Fragile X syndrome, Angelman syndrome, Tuberous sclerosis complex, Neurofibromatosis type 1, Autism spectrum disorder, Latent profile analysis

## Abstract

Studying Autism Spectrum Disorder (ASD) heterogeneity in biologically homogeneous samples may increase our knowledge of ASD etiology. Fragile X syndrome (FXS), Angelman syndrome (AS), Tuberous Sclerosis Complex (TSC), and Neurofibromatosis type 1 (NF1) are monogenic disorders with high a prevalence of ASD symptomatology. This study aimed to identify ASD symptom profiles in a large group of children and adolescents (0;9–28 years) with FXS, AS, TSC, and NF1. Data on ASD symptomatology (Autism Diagnostic Observation Scale (ADOS-2) & Social Responsiveness Scale (SRS-2)) were collected from children and adolescents with FXS (*n* = 54), AS (*n* = 93), TSC (*n* = 112), and NF1 (*n* = 278). To identify groups of individuals with similar ASD profiles, we performed two latent profile analyses. We identified a four-profile model based on the ADOS-2, with a (1) ‘Non-spectrum symptom profile’, (2) ‘Social Affect symptom profile’, (3)‘Restricted/Repetitive Behaviors symptom profile’, and (4)‘ASD symptom profile’. We also identified a four-profile model based on the SRS, with a (1)‘Non-clinical symptom profile’, (2)‘Mild symptom profile’, (3)‘Moderate symptom profile’, and (4)‘Severe symptom profile’. Although each syndrome group exhibited varying degrees of severity, they also displayed heterogeneity in the profiles in which they were classified. We found distinct ASD symptom profiles in a population consisting of children and adolescents with FXS, AS, TSC, and NF1. Our study highlights the importance of a personalized approach to the identification and management of ASD symptoms in rare genetic syndromes. Future studies should aim to include more domains of functioning and investigate the stability of latent profiles over time.

**Supplementary Information:**

The online version contains supplementary material available at 10.1007/s10803-024-06557-2.

Autism Spectrum Disorder (ASD) is known for the large heterogeneity in the symptoms that individuals experience, as well as heterogeneity in the genetic origin of these symptoms (Rodriguez-Fontenla & Carracedo, 2021; Tick et al., 2016). Monogenic disorders with a high ASD prevalence are often studied to gain insight into the etiology of ASD and its symptomatology (Bozhilova et al., 2023; Frewer et al., 2021; Moss & Howlin, 2009). As these groups are more homogeneous on a neurobiological level, studying the ASD symptomatology allows for understanding the genetic and neurobiological underpinnings. Insight into these pathways could reveal (neural) biomarkers that could serve as indicators for treatments and early interventions. Examples of monogenic syndromes with a high prevalence of ASD symptomatology are Fragile X Syndrome (FXS), Angelman Syndrome (AS), Tuberous Sclerosis Complex (TSC), and Neurofibromatosis Type 1 (NF1) (Richards et al., 2015). In all these syndromes the mechanistic target of rapamycin (mTOR) pathway is dysregulated (Bagni & Zukin, 2019; Fahnestock & Nicolini, 2015; Feliciano, 2020; Henske et al., 2016; Kelleher & Bear, 2008; Lodish & Stratakis, 2010; Maranga et al., 2020; Millan, 2013), which has also been found in non-syndromic ASD (Winden et al., 2018).

In a previous study conducted by our group, we compared ASD symptomatology in children and young adults with FXS, AS, TSC, and NF1 (Lubbers et al., 2022). These four monogenic syndromes were compared on a group-level based on their scores on two ASD assessment tools: the Autism Diagnostic Observation Schedule (ADOS (Lord et al., 1999) and the Social Responsiveness Scale (SRS (Constantino & Gruber, 2012). Overall, the study found that ASD symptomatology was most severe in individuals with FXS and least severe in those with NF1. Additionally, the study identified specific strengths and challenges associated with each syndrome. For instance, the domain of restrictive and repetitive behavior presented a relative challenge for FXS and, to a lesser extent, TSC, but was relatively unaffected in those with NF1. The study found that children with AS demonstrated relative strengths in the social communication and motivation domains, but faced challenges in reciprocal social interaction and in the creativity and play scales of the ADOS. While this study provided valuable information about the variability in symptom presentation between syndrome groups, it is also in line with other results (Bozhilova et al., 2023) showing that significant variability can be found within syndrome groups.

Unlike hypothesis-driven methods that compare ASD symptomatology between syndrome groups, unsupervised learning methods can reveal subgroups of individuals within a sample with shared genetic variants based on similarities in their ASD symptomatology. One example of such an unsupervised learning approach is latent profile analysis (LPA). LPA is used to model latent categorical profiles in which individuals are probabilistically classified within a profile based on the variables used in the model (Spurk et al., 2020). Individuals in the same profile score similarly on the indicator variables of the model. This method has previously been used to identify subgroups among individuals with ASD across several behavioral domains, including aggressive behavior (Sullivan et al., 2019), quality of life (Azad et al., 2020), restrictive and repetitive behavior (Zheng et al., 2019), and autistic traits (James et al., 2016) (for a review, see (van Agelink et al., 2021). The studies’ subgroups highlight that ASD cannot be classified on a linear spectrum, with all symptoms being more severe in individuals with an ASD classification than in those without. Instead, ASD requires a multidimensional approach, because various patterns of symptom presentation are observed, in which symptom severity varies for different domains of symptoms. Furthermore, LPA has been utilized to demonstrate that phenotypical subgroups, based on multiple cognitive and behavioral domains, within a population affected with ASD exhibit varying responses to treatment (Préfontaine et al., 2022). Therefore, studying phenotypical ASD subgroups is crucial for enhancing our comprehension of ASD symptomatology and improving treatment efficacy.

The objective of this study is to use an unsupervised learning approach to identify subgroups with a similar ASD symptom profile based on ADOS and SRS data within a sample of children and adolescents with FXS, AS, TSC, and NF1. The study will focus on the two main symptom domains of ASD: the social affect domain (SA) and the restrictive/repetitive behavior (RRB) domain, which are the two outcome domains of the ADOS. For the ADOS analyses, we anticipate finding subgroups with the following profiles: (1) normal social functioning and low repetitive behavior, (2) low social functioning and low repetitive behavior, (3) normal social functioning and high repetitive behavior, and (4) low social functioning and high repetitive behavior. Since the SRS has 5 outcome domains, we do not have any prior hypotheses about the number or shapes of the latent SRS profiles. For the four included syndromes, we already know that the variability in ASD symptom presentation, between and within syndrome groups, may be partly explained by well-known factors, such as the genetic component (Lubbers et al., 2022; Richards et al., 2015), the intellectual ability of individuals (Wolff et al., 2022), gender differences (Rinehart et al., 2010; Rivet & Matson, 2011), and the presence of epilepsy (Bernardo et al., 2020; Berry-Kravis, 2002; Moavero et al., 2016; Thibert et al., 2009). In a descriptive way, we will therefore also explore how syndrome type, age, gender, developmental level, and epilepsy are related to the identified profiles.

Focusing on subgroups with specific symptom profiles across monogenic syndrome groups provides insight into the extent to which symptom presentation varies across these monogenic disorders and into the factors that may contribute to this variation. Clinicians can use these profiles to provide more person-centered information and develop targeted treatment plans that address the needs of individuals within specific subgroups (Préfontaine et al., 2022). The identification of distinct patterns of symptom presentation in children with monogenic disorders may also contribute to the understanding of the etiology of ASD and to the search for (neural) biomarkers of ASD, which are crucial in optimizing diagnostics and treatment (van Agelink et al., 2021).

## Methods

### Participants

Our sample consists of children and young adults, aged 0; 9–28 years, with syndromes that have a high prevalence of ASD symptomatology: FXS, AS, TSC, and NF1 (see Table 1 for sample characteristics). All children were assessed for ASD symptoms and cognitive functioning as part of the routine clinical care provided to all children seen at the ENCORE Expertise Center for neurodevelopmental disorders within the Erasmus MC Sophia Children’s Hospital in Rotterdam, the Netherlands. All children with these syndromes were included, regardless of behavioral/emotional symptomatology.

**Table 1:** Sample characteristics

		FXS	AS	TSC	NF1
ADOS (N = 537)					
N		54	93	112	278
Age					
M (SD)		8.02 (5.4)	7.7 (4.3)	8.4 (4.9)	8.0 (4.2)
range (y)		2–28	2–21	2–19	1–18
Males n (%)		40 (74)	48 (52)	59 (53)	155 (56)
ASD classification n (%)		36 (66.7)	13 (14.0)	52 (46.4)	39 (14.0)
IQ/DQ					
M (SD)		51.2 (18.4)	20.5 (13.9)	62.4 (29.2)	85.5 (16.7)
range		20.0–93.0	3.2–68.0	4.0-127.0	26.0-135.0
ADOS module					
Module 1 N(%)		26 (48.1%)	92 (98.9%)	35 (31.3%)	23 (8.3%)
Module 2 N(%)		14 (25.9%)	-	17 (15.1%)	57 (20.5%)
Module 3 N(%)		12 (22.2%)	1 (1.1%)	43 (38.4%)	191 (68.7%)
Module 4 N(%)		2 (3.7%)	-	17 (15.2%)	7 (2.5%)
SRS (N = 465)					
N		50	53	97	265
Age					
M (SD)		8.1 (5.1)	9.0 (4.7)	9.7 (5.2)	7.0 (3.4)
range (y)		0;9–26	2–20	1–19	2–17
Males N (%)		36 (72)	30 (57)	48 (49)	146 (55)
ASD classification n (%)		31 (62.0)	10 (18.9)	44 (45.4)	35 (13.2)
IQ/DQ					
M (SD)		52.4 (21.4)	17.3 (10.5)	64.0 (29.4)	86.0 (16.7)
range		9.0-111.0	3.2–52.2	4.0-127.0	26.0-135

*Note*. FXS = Fragile X Syndrome, AS = Angelman Syndrome, TSC = Tuberous Sclerosis Complex, NF1 = Neurofibromatosis Type 1, DQ = Developmental Quotient, y = years

### Measures

To assess the presence and severity of ASD symptoms, we used the Autism Diagnostic Observation Schedule (ADOS) and the Social Responsiveness Scale (SRS). These instruments are often used together in clinical practice as they provide insight into a child’s behavior from different perspectives.

#### ADOS

The ADOS is a semi-structured schedule of activities that allows researchers or clinicians to observe an individual’s behavior in areas associated with a diagnosis of ASD: social communication, reciprocal social interactions, and restricted and repetitive behavior. We used the Social Affect (SA) and Restricted and Repetitive behavior (RRB) subscales for our ADOS analyses. The sensitivity of the ADOS-2 ranges from 72 to 97% and the specificity from 19 to 94%, depending on the module used (Gotham et al., 2007) and the developmental level of the child (Miller et al., 2019).

Data were collected using the ADOS-G (5.4%) (Lord et al., 1999) and the ADOS-2 (94.6%) (Lord, 2012). All assessments were carried out by formally trained and certified psychologists of the department of child and adolescent psychiatry/psychology at the Erasmus MC Sophia Children’s Hospital in Rotterdam, the Netherlands. ADOS-G scores were converted to ADOS-2 scores using the manual (Lord, 2012). The ADOS provides a calibrated severity score (CSS) that represents the severity of ASD symptomatology on a scale of 0–10, with higher scores indicating greater symptom severity. Based on the total CSS, individuals are classified as ‘non-spectrum’ (scores 1–3), ‘ASD’ (scores 4–5) or ‘Autism’ (scores > 5) (Hus et al., 2014; Hus & Lord, 2014). One of five available modules is selected based on age and verbal abilities. In addition, the scores are corrected for the chronological age of the individual. However, norm groups are not available for children with a severe developmental delay. Therefore, for children older than the available norm groups we calculated scores based on the nearest available norm group. The calibrated severity scores (CSS) for the SA and RRB domains were used in our analysis. As in our previous study (Lubbers et al., 2022), we used the untransformed RRB subscale score (range 0–7) in our analyses instead of the transformed RRB CSS (range 1–10, where values 2, 3, and 4 are not possible). The transformed CSS improves the clinical interpretation of ADOS scores, but is problematic for statistical analysis (Abbeduto et al., 2019).

#### SRS

The SRS is a 65-item ASD screening questionnaire, on which parents, caregivers, or teachers rate a child’s behavior over the past six months on a 4-point Likert scale. The items are grouped into five subscales: Social Awareness, Social Cognition, Social Communication, Social Motivation, and Autistic Mannerisms. Age- and gender-normed T-scores can be calculated for the total score and for the domains ‘Social Communication and Interaction’ (consisting of the first four subscales) and ‘Restricted Interests and Repetitive Behavior’ (consisting of the Autistic Mannerisms subscale), with higher scores indicating greater symptom severity. In addition, T-scores can be calculated for all subscales. Based on the T-scores, ASD symptom severity is interpreted as non-clinical (T < 60), mild (60 ≤ T ≤ 65), moderate (65 ≤ T ≤ 75), or severe (75 ≤ T) (Bruni, 2014). The sensitivity of the SRS-2 ranges from 74 to 80% and the specificity from 85 to 90% (Bölte et al., 2011). The dataset includes data from both the SRS and SRS-2. As items do not differ between the SRS and the SRS-2, scores were classified using the SRS-2 classification methods and norm tables, regardless of the questionnaire used. For children younger or older than the available norm groups, we calculated scores based on the closest available norm group.

#### Cognitive Functioning

Cognitive functioning, represented by IQ or Developmental Quotient (DQ) scores, was included as a descriptive variable of the latent profiles, as developmental levels differ between syndrome groups and individuals within these groups. As children with a developmental delay are more likely to score in the clinical range on both ADOS (Molloy et al., 2011) and SRS (Hus et al., 2013), differences in cognitive functioning within and between groups may influence our results. Cognitive functioning was assessed using an age- or developmentally appropriate instrument. Instruments included the Wechsler Non Verbal scale of Ability (WNV) (Wechsler, 2006), the Snijders-Oomen Non-verbal intelligence test (SON) (Tellegen, 2011), the Bayley Scales of Infant and Toddler Development third edition (Bayley-III) (Bayley, 2006), Wechsler Preschool and Primary Scale of Intelligence (WPPSI-III-NL (Wechsler, 2002) or WPPSI-IV-NL (Wechsler, 2012), the Wechsler Intelligence Scale for Children (WISC-III-NL (Wechsler, 1991) or WISC-V-NL (Wechsler, 2014), the Wechsler Adult Intelligence Scale (WAIS-III) (Wechsler, 1997), and the Vineland Screener (Sparrow et al., 1993) (only if an assessment failed). In some cases, it was not possible to calculate an IQ score because the chronological age of the individual was higher than the available norm groups (e.g. when the WPPSI was used to assess a 16-year-old with developmental delay). In these cases, a DQ score was calculated instead (DQ = [estimated developmental age divided by chronological age] times 100, with M = 100, SD = 15) (van Eeghen et al., 2012).

#### ASD Classification Status

ASD classification status, based on DSM-IV (American Psychiatric Association, 2000) and DSM-V (American Psychiatric Association, 2013) criteria, was retrieved from our participant’s patient records. Classifications were based on clinical evaluation by a team including a psychiatrist and psychologists from the department of child and adolescent psychiatry/psychology at the Erasmus MC Sophia Children’s Hospital in Rotterdam, the Netherlands. The following DMS-IV classifications were considered as an ASD classification: autistic disorder, Asperger’s disorder, pervasive developmental disorder – not otherwise specified.

### Procedure

The study procedures were pre-registered on OSF (https://osf.io/pb5r4). The data of the syndromic groups consist of assessments of individuals with a complete ADOS and/or SRS assessment between May 2009 and July 2022. Individuals without an available IQ/DQ score were excluded. To ensure that the most recent versions of the assessment instruments were included whenever possible, the most recent complete assessment was selected if data were available from multiple time points for an individual. In the case where both teacher and caregiver reports were available for the SRS, we included the caregiver’s report in our analysis.

### Statistical Analysis

Statistical analyses were performed in R (R Development Core Team, 2022). Our analysis script is made publicly available at https://osf.io/tdn6h/.

#### Primary Variables

We used the ADOS CSS-SA and the untransformed CSS-RRB (hereafter CSS-RRB) to fit the first LPA and k-means models and the five SRS subscale T-scores (Social Awareness, Social Cognition, Social Communication, Social Motivation, and Autistic Mannerisms) to fit the second LPA and k-means models.

#### LPA Modelling

We started by removing multivariate outliers and checking the distribution of the ADOS and SRS scores. We removed multivariate outliers based on their Mahalanobis distance using the *rstatix* package (Kassambra, 2021). We did not expect to find a normal distribution in the data, but we visually checked for extreme skewness. We then used the *Mclust* package (Scrucca et al., 2016) to fit a model based on the indicator variables for both the ADOS and SRS data separately. *Mclust* uses an expectation maximization–algorithm to estimate the best-fitting model with *n* – profiles. Since there is no consensus how to choose the best-fitting LPA model (Spurk et al., 2020), our model selection was based on an integrative evaluation of the AIC, BIC, SABIC, entropy, likelihood ratio test, and clinical interpretability. We used a multivariate analysis of variance on the indicator variables to assess whether the means of the indicator variables differed between the profiles. We described each profile’s characteristics based on age, sex, syndrome group, epilepsy, and IQ/DQ.

Hereafter, we repeated the LPA modelling of the ADOS and SRS data once for the NF1 group only and once for the FXS, AS, and TSC groups combined. First, this gave us insight into the sensitivity of our LPA model from the main analysis. Secondly, performing the analysis in a sample of NF1-only patients gave insight into the degree of heterogeneity in an etiologically homogenous subgroup. We are not yet able to perform LPAs for the remaining syndrome groups individually, as our sample sizes are insufficient.

#### K-means Clustering

We used k-means clustering to establish the methodological validity of the LPA model we found (Grant et al., 2020; Liu et al., 2022; Spurk et al., 2020). In doing so, we tested the stability of the profile classification from the LPA by comparing it to another unsupervised classification algorithm. We used the *cluster* package (Maechler et al., 2021) to perform our k-means analysis. K-means clustering requires a pre-specified number of clusters. This number is usually determined based on previous studies or the calculation of multiple indices. We used the number of profiles found in the LPA modelling step. However, we also used the *nbclust* function (Charrad et al., 2014) to calculate 30 goodness-of-fit indices that indicate whether our choice of *k* is appropriate. Finally, we performed k-means clustering with 1000 random initial starting points. We then qualitatively compared LPA and k-means solutions based on their profile/cluster means for each variable. The results of the k-means clustering validation are presented in the supplementary materials.

#### Exploratory Analyses

We conducted a multinomial logistic regression analysis to investigate whether the latent group classification from our LPA model was associated with age, sex, syndrome group, epilepsy, or IQ/DQ. The results from the exploratory analyses are reported in the supplementary material.

## Results

### Descriptives

Sample characteristics are presented in Table 1. A total of 552 children were included in our study. ADOS data were available for 537 children (56% male, no outliers, mean age (SD) = 8.0 (4.4) years) and SRS data were available for 465 children (56% male, 6 outliers removed, mean age (SD) = 7.9 (4.3) years). The largest syndrome group consisted of children with NF1 (51.6%), followed by TSC (20.8%), AS (16.8%), and FXS (10.7%).

### Model Selection

We performed an LPA for the ADOS with CSS-SA and CSS-RRB as indicator variables and an LPA for the SRS with the five SRS subscale T-scores as indicator variables. For each analysis, we compared models with one to five profiles based on fit statistics and clinical interpretability. The model fit statistics are presented in Table S1.

### ADOS

#### Latent Profile Analysis

All fit statistics support a five-profile model for the ADOS data. However, the five-profile model results in two profiles that cannot be interpreted as meaningfully different from each other, as their mean scores for both ADOS domains fall in the same CSS classification ranges (Hus et al., 2014; Hus & Lord, 2014). Given the clinical relevance, we chose the more parsimonious four-profile model as our final solution. The posterior classification probabilities of the four-profile model are well above the 0.80 threshold (Spurk et al., 2020), ranging from 0.86 to 0.99.

**Fig. 1:**
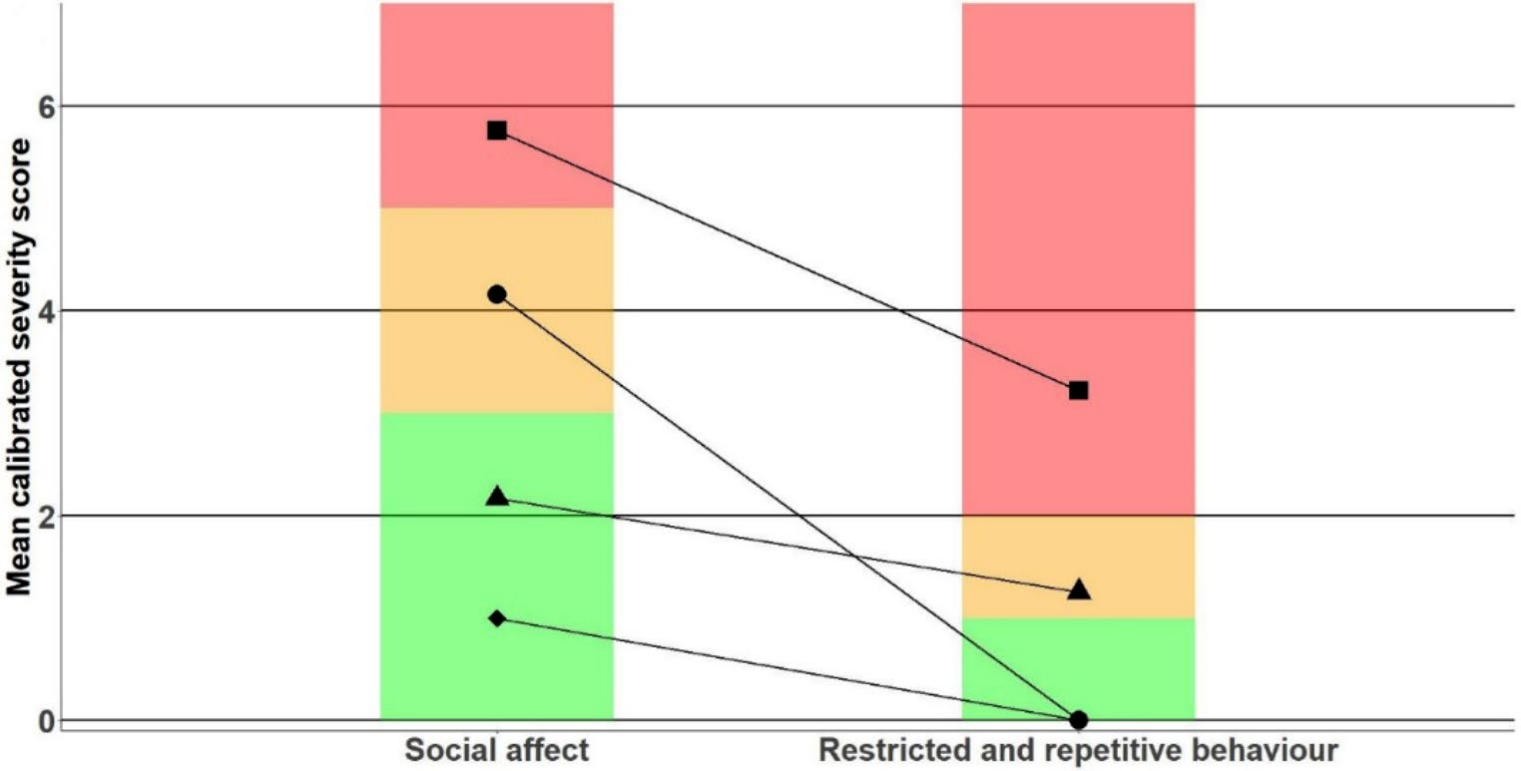
Profile plots of the LPA ADOS model. ♦ = non-spectrum profile, ● = SA profile, ▲ = RRB profile, ■ = ASD profile. Green indicates symptoms in the non-clinical range, orange in the ASD range, and red in the autism range (Lord, 2012)

A MANOVA with profile assignment as predictor shows significant differences between the profiles on the CSS for both the SA and RRB domains (*F*(3,533) = 184.21, *p* < .001, *η*^*2*^_*p*_ = 0.51). The profiles are interpreted as follows: a non-spectrum profile (*n* = 83, 39.8% male, 7.2% ASD classification, mean IQ = 85.3, epilepsy rate = 14.5%), an RRB profile (*n* = 130, 51.5%, 7.7% ASD classification, male, mean IQ = 74.9, epilepsy rate = 26.9%), an SA profile (*n* = 120, 53.3% male, 15% ASD classification, mean IQ = 78.5, epilepsy rate = 27.5%), and an ASD profile (*n =* 204, 67.4% male, 48% ASD classification, mean IQ = 45.1, epilepsy rate = 51.9%). Figure 1 depicts the profile shapes. Individuals in the non-spectrum profile show non-spectrum symptom severity in both the SA and RRB domains. Individuals in the RRB and SA profiles show ASD symptoms on the autism spectrum level in the SA and RRB domains, respectively, while scoring within the normal range in the other domain. Finally, individuals in the ASD profile have scores in the autism range on both the SA and RRB domains. Table 2 presents summary statistics for the ADOS CSSs and descriptive statistics per profile. Although k-means cluster analysis resulted in some differences compared to our identified profiles, we found roughly the same subgroups which indicates methodological validity of our LPA model (see Supplementary Materials).

Most individuals were classified within the ASD profile (37.9%), followed by the RRB profile (24.2%), the SA profile (22.3%), and the non-spectrum profile (15.5%) (see Table 2). Syndrome group was significantly related to profile assignment (Χ²(9) = 154.67, *p* < .001). Participants with NF1 are less likely to be assigned to the ASD profile and more likely to the non-spectrum, RRB, and SA profiles, while children with AS and FXS are more likely to be assigned to the ASD profile and less likely to the non-spectrum, RRB, and SA profiles. Individuals with TSC are more likely to be assigned to the RRB, SA, and ASD profiles compared to individuals with NF1, but less likely compared to individuals with FXS or AS.

**Table 2:** Summary statistics of the ADOS profiles

*ADOS profiles*	CSS-RRB		CSS-SA	Age (y)	Male/Female (n)	ASD classification (%)	IQ/DQ	Epilepsy (%)
Non-spectrum	0.0		1.0	7.3	33/50	7.2	85.3	14.5
RRB	1.3		2.2	7.7	67/63	7.7	74.9	26.9
SA	0.0		4.2	7.6	64/56	15.0	78.5	27.5
Autism	3.2		5.8	8.8	138/66	48.0	45.1	51.9
		FXS (n(%))			AS (n(%))	TSC (n(%))	NF1 (n(%))	Χ²
*Profiles*								154.67*
Non-spectrum		0 (0)			4 (4.3)	10 (8.9)	69 (24.8)	
RRB		5 (9.2)			16 (17.2)	22 (19.6)	87 (31.3)	
SA		3 (5.6)			10 (10.8)	30 (26.8)	77 (27.7)	
Autism		46 (85.2)			63 (67.7)	50 (44.6)	45 (16.2)	

*Note*. CSS-RRB = Restrictive and Repetitive Behavior Calibrated Severity Score, CSS-SA = Social Affect Calibrated Severity Score, y = years, DQ = Developmental Quotient, FXS = Fragile X Syndrome, AS = Angelman Syndrome, TSC = Tuberous Sclerosis Complex, NF1 = Neurofibromatosis Type 1, *p < .001

### SRS

#### Latent Profile Analysis

The BIC supports a three-profile model, while the SABIC, AIC, entropy, and the BLRT support a four-profile model. We chose the four-profile model as our final model because the majority of the fit statistics favor this model and all profiles can be interpreted as meaningfully different from each other (Berlin et al., 2014; Spurk et al., 2020). The posterior classification probabilities ranged from 0.82 to 0.89, which is above the recommended threshold of 0.80 (Spurk et al., 2020). Summary statistics of the SRS T-scores and descriptives are presented in Table 3.

**Fig. 2:**
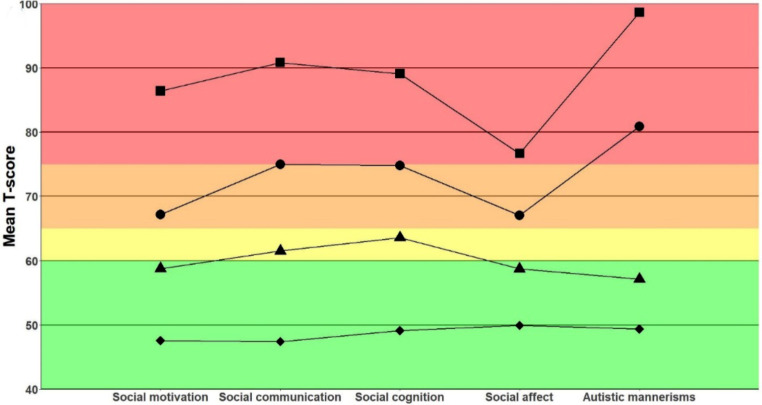
(**a**) Profile plots of the LPA SRS model. (**b**) Comparison of the LPA SRS model and K-means SRS model. ■ = severe profile, ● = moderate profile, ▲ = mild profile, ♦ = non-clinical profile. Green indicates symptoms in the non-clinical range, yellow in the mild range, orange in the moderate range, and red in the severe range (Bruni, 2014)

A MANOVA showed that the profiles were significantly different on the SRS T-scores (*F*(2,462) = 103.42, *p* < .001, *η*^*2*^_*p*_ *=* 0.37). The profiles are best interpreted as a non-clinical symptom profile (*n =* 160, 52.5% male, 5.6% ASD classification, mean IQ = 89.0, epilepsy rate = 6.9%), a mild symptom profile (*n =* 130, 60.8% male, 17.7% ASD classification, mean IQ = 75.4, epilepsy rate = 28.5%), a moderate symptom profile (*n =* 156, 59.6% male, 48.1% ASD classification, mean IQ = 46.9, epilepsy rate = 54.5%), and a severe symptom profile (*n* = 19, 21.1% male, 68.4% ASD classification, mean IQ = 53.7, epilepsy rate = 36.8%) (see Table 3). Individuals in the non-clinical symptom profile have T-scores within the non-clinical range. For the mild symptom profile, individuals have T-scores in the mild symptom range for the social cognition and communication domains, but non-clinical T-scores for the other domains. In the moderate symptom profile, individuals have severe symptoms in the autistic mannerisms domain and moderate symptoms in all other domains. The severe symptom profile is characterized by severe symptoms in all domains. The profile shapes are depicted in Fig. 2. A k-means cluster analysis revealed the same subgroups as our LPA model (see supplementary material).

Most individuals are classified in the non-clinical symptom profile (34.4%), followed by the moderate symptom profile (33.5%), the mild symptom profile (28.0%), and the severe symptom profile (4.1%). Again, syndrome group was significantly related to profile assignment (Χ²(9) = 189.87, *p* < .001). Individuals with NF1 were more likely to be assigned to the non-clinical profile and less likely to be assigned to the moderate and severe profiles. Individuals with FXS, AS, or TSC were less likely to be assigned to the non-clinical profile and more likely to be assigned to the moderate and severe profiles.

**Table 3:** Summary statistics of the SRS profiles

*SRS Profiles*	SA	SCog	SCom	SM	AM	Male/Female (n)	ASD classification (%)	Age (y)	IQ/DQ	Epilepsy (%)
Non-clinical	50.0	49.1	47.2	47.6	49.4	84/76	5.6	6.6	89.0	6.9
Mild	58.7	63.6	61.5	58.8	57.1	79/51	17.7	8.1	75.4	28.5
Moderate	67.1	74.8	75.0	67.2	80.9	93/63	48.1	8.6	47.9	54.5
Severe	76.7	89.2	90.8	86.4	98.6	4/15	68.4	11.6	53.7	36.8
		FXS (n(%))		AS (n(%))		TSC (n(%))		NF1 (n(%))		Χ²
*Profiles*										189.877*
Non-clinical			2 (4.0)		0 (0.0)	11 (12.3)		147 (55.5)		
Mild			12 (24.0)		9 (17.0)	34 (38.2)		75 (28.3)		
Moderate			28 (56.0)		43 (81.1)	43 (48.3)		42 (15.8)		
Severe			8 (16.0)		1 (1.9)	1 (1.1)		1 (0.4)		

*Note*. SA = social affect, SCog = social cognition, SCom = social communication, SM = social motivation, AM = autistic mannerisms, y = years, DQ = Developmental Quotient, FXS = Fragile X Syndrome, TSC = Tuberous Sclerosis Complex, AS = Angelman Syndrome, NF1 = Neurofibromatosis Type 1, *p < .001

### Exploratory Analyses

#### Subgroup Analyses

We repeated the ADOS and SRS LPAs once for the NF1 group alone, and once for the FXS, AS and TSC groups combined (see supplementary material*)*. We found that the LPA profiles for the NF1 group were similar to the profiles for both of the main analyses, except that the symptom severity of the profiles in the NF1 analyses were lower compared to the main analyses. Additionally, when we combined the FXS, AS, and TSC groups, we found similar profiles as in both of the main analyses, but these profiles showed higher symptom severity compared to the main analyses. Combined, these results show that the interpretation of the relative strengths and weakness of the profiles does not change drastically when conducting subgroup analyses. Finally, the LPAs for NF1 group support that ASD symptoms manifest heterogeneously, despite genetic homogeneity.

#### Predictors of Profile Assignment

For the ADOS profiles, higher IQ/DQ scores are related to a lower chance of being assigned to the ASD profile (Table S2 for test statistics). Females tend to have a lower symptom severity: they have a lower chance of being in the ASD, RRB, or SA profile. Since we found very high ORs for the effect of syndrome group, we don’t interpret these ORs here. The NF1 group seems to be the least affected group based on the distribution across profiles. Age was not related to any symptom profile.

For the SRS profiles, higher IQ/DQ scores are related to a lower chance of having severe, moderate, or mild symptoms (Table S3 for test statistics). Females seem more severely affected compared to the ADOS, but the wide 95% CI reflects profile imbalance. Older children are more likely to experience more severe ASD symptoms. Children with TSC, FXS, or AS are all more likely to have severe, elevated, or mild symptoms compared to NF1. Even though the ORs are inflated, we can still conclude that the NF1 group is less affected than the remaining syndrome groups.

## Discussion

The aim of this study was to identify latent ASD symptom profiles based on two assessments, which differ in method and information source, in a heterogeneous sample of children and adolescents with FXS, AS, TSC, and NF1. The ADOS is an assessment carried out by a trained clinician in a structured setting, while the SRS is a questionnaire filled out by parents, caregivers or teachers of the children and assesses a child’s behavior in daily life. We fitted LPA models based on the ADOS CSS and SRS T-scores, resulting in profiles that differed in symptom type and severity for the ADOS data and profiles that differed in symptom severity for the SRS data. As hypothesized, we found ADOS profiles with (1) normal social functioning and low repetitive behavior (non-spectrum profile), (2) low social functioning and low repetitive behavior (SA profile), (3) normal social functioning and high repetitive behavior (RRB profile), and (4) low social functioning and high repetitive behavior (ASD profile). We also found four SRS profiles, ranging from non-clinical to severe symptoms. These results stress the heterogeneity of ASD symptoms. Although individuals with NF1 were less severely affected compared to individuals with FXS, AS, and TSC, individuals within specific syndrome groups were distributed across multiple profiles.

Our subgroup analyses showed that the profile severity, but not the interpretation of the profile, is sensitive to the groups we included in our analyses. The subgroup analysis of the NF1 group showed that ASD symptoms manifest in a heterogeneous manner, even in an etiologically homogeneous group. Our analysis of the predictors of profile assignment indicate that cognitive functioning may be related to profile severity. Specifically, children with lower cognitive functioning are overrepresented in the more severely affected profiles. Again, interpretation of our predictor analysis should be done with care due to the inflated ORs.

The findings of our study are in line with both the severity gradient and distinct subgroup frameworks of ASD symptoms (Syriopoulou-Delli & Papaefstathiou, 2020). In their systematic review, Syriopoulou-Delli and Papaefstathiou (2020) found that most studies investigating subgroups in non-syndromic ASD populations found evidence for the severity gradient theory of ASD. That is, we can distinguish subgroups based on ASD symptoms, but these subgroups are distinguishable only on symptom severity and not on specific symptom domains. Indeed, we found SRS profiles that only differed in symptom severity, supporting the symptom severity framework. However, Syriopoulou-Delli and Papaefstathiou (2020) also found several studies supporting the distinct subgroup framework (or both frameworks). Our ADOS analysis is in line with both frameworks: we did find subgroups with weaknesses in specific domains but also subgroups that differ in severity. Both frameworks might hold some truth (Syriopoulou-Delli & Papaefstathiou, 2020) and our mixed results stress the need to incorporate information from multiple sources when identifying latent symptom profiles.

### Syndrome-Specific Findings

Considering individual syndromic groups, starting with FXS, the current literature suggests that most children with FXS experience ASD-related symptoms (Bailey et al., 2000; Marlborough et al., 2021; McDuffie et al., 2015; Wheeler et al., 2015), whereas 15 to 36% of individuals with a full FXS mutation score within the clinical range based on ASD assessment instruments. Consistent with the fact that most children with FXS experience ASD-related symptoms, we found that almost none of the children with FXS in our sample fall within the non-clinical LPA profiles.

According to the literature, most children with AS have ASD symptoms (Leader et al., 2022; Trillingsgaard & ØStergaard, 2004), with ASD-classification rates for children with AS ranging from 20 to 80% (Bonati et al., 2007; Leader et al., 2022; Richards et al., 2015; Sahoo et al., 2006; Trillingsgaard & ØStergaard, 2004; Veltman et al., 2005). Consistent with this, our ADOS analyses show that most children with AS fall within the most severe profile, and the SRS analysis shows that most children fall within the moderate symptom profile. There is also evidence in the literature that children with AS show relative strengths within the social domain (Lubbers et al., 2022; Trillingsgaard & ØStergaard, 2004). In our ADOS analyses, 21% of our sample of children with AS were classified in profiles with a relatively low level of symptoms within the social domain. Although this is a substantial group, the majority of individuals are classified in profiles showing problems in the social domain. As our analyses were conducted at the subscale level, we cannot confirm nor deny whether this relative strength may exist at the item level.

For individuals with a TSC mutation, a score within the clinical range based on ASD assessment instruments is reached by approximately 35 to 60% (Curatolo et al., 1991; de Vries et al., 2007, 2020; Jeste et al., 2016; Lubbers et al., 2022; Richards et al., 2015; van Eeghen et al., 2013). While most TSC studies focus on the social domain rather than the RRB domain, our previous study showed that individuals with TSC show a relatively high degree of symptom severity within the RRB domain (Lubbers et al., 2022). In the current study the results of the ADOS analysis support the high prevalence of ASD symptoms, with almost half of the children falling within the most severe profile. We highlight the fact that ASD symptoms manifest in a more heterogeneous manner than might be expected from the current literature on ASD symptomatology in TSC: almost all children with TSC who do not fall within the most severe profile show impairments on either the social or restrictive and repetitive behavior domain. In addition, the SRS analysis showed that the vast majority of the children show ASD symptoms to some extent, with half of the children falling within the two most severe profiles.

Children with NF1 have relatively low rates of ASD compared to the other syndromes, ranging from 10 to 39% (Eijk et al., 2018; Lubbers et al., 2022; Morris et al., 2016; Richards et al., 2015; van Eeghen et al., 2013). Indeed, in both the ADOS and SRS analyses, the least affected profiles consisted mostly of children with NF1. However, the ADOS analysis indicated that 75% of our NF1 sample showed clinically relevant symptoms in at least one domain. On the other hand, the SRS analysis showed a large majority of the children with NF1 in the two least affected profiles. These mixed results were also found for the NF1 subgroup analysis.

### Effects of Assessment Method on Measured ASD Symptomatology

We found substantive differences in our results between the ADOS and SRS analyses. The SRS is a questionnaire filled out by parents, caregivers or teachers of the children and assesses a child’s behavior in daily life, while the ADOS is an assessment carried out by a trained clinician in a structured setting. As these assessments are based on different information sources and also measure different types of information, this may have led to the differences in our results. A study in individuals with FXS (Fielding-Gebhardt et al., 2021) showed that different outcome measures lead to different ASD classifications. Indeed, we found a discrepancy between outcome measures for all investigated syndromes. Our findings stress the importance of considering multiple sources of information while making an ASD diagnosis.

Another factor that might have driven the differences in our results is the adaptability of the assessments to suit the cognitive level of an individual. There is discussion in the field about whether children with developmental delay should be considered for a DSM-based ASD classification or whether their ASD symptoms should be solely attributed to their lower level of cognitive functioning. Indeed, we did find that individuals in the more severe profiles generally showed a lower level of cognitive functioning. In addition, even in the most severely affected profiles, ASD classification status was not higher than 50%. As the ADOS assessment can be adapted by the administrator to suit the developmental level of the individual, it might be expected that a low developmental level should not necessarily result in a higher ADOS CSS. Since the revision of the ADOS algorithms, sensitivity has improved for modules 2 and 3, however this effect has not been observed for module 1 because of the lower cognitive functioning of the children assessed with this module (de Bildt et al., 2009; Medda et al., 2019). Even the adjustability of the ADOS is limited to the activities of ADOS module 1, after which there is no possibility of switching to a less cognitively demanding module. Similarly, the adaptability of the SRS is limited as even in the version for the lowest possible developmental level, some questions may still not be applicable to the assessed individual. Because of a lack of contextual information, high scores on the SRS might be indicative of a developmental delay instead of, or as well as an ASD. Therefore, when interpreting scores on an ASD assessment or screening tool a possible developmental delay of a child should always be taken into account. In our sample, this effect of cognitive development on ADOS and SRS scores might have had particularly strong effect for the children with AS, as they had the most severe developmental delay compared to the other syndrome groups. Indeed, for all children with AS except one ADOS module 1 was administered. The one child that was administered with a higher module had mosaic AS, which is characterized by a milder symptom profile (Le Fevre et al., 2017).

### Strengths and Limitations

Our study has multiple strengths. First, we validated our results using k-means cluster analysis. The ADOS LPA profiles differ somewhat from the ADOS k-means clusters. These differences are likely due to the fact that LPA and k-means clustering use different algorithms to classify participants. LPA is a Gaussian finite mixture modelling approach assuming that individuals can be classified into a latent subgroup with a certain probability (Spurk et al., 2020), whereas k-means clustering is a model-free classifier (Li & Wu, 2012). Even though the results of the analyses methods showed minor differences, the direction and shapes of the clusters were similar to those of the LPA profiles, and therefore we still deemed our model to be valid. The interpretation of the SRS clusters is highly similar to the SRS LPA profiles, which indicates methodological stability of our SRS LPA model. Second, we obtained a relatively large and well-characterized sample size for these rare genetic disorders. Selection bias is minimal as data was collected through routine clinical care. For FXS however, somatic problems are generally limited and therefore it might be the case that only individuals with more severe behavioral problems choose to visit the ENCORE center of expertise. This means that individuals with FXS with milder behavioral problems, especially females who are generally less severely affected than males, may be underrepresented in our sample. Additionally, although our sample size was insufficient to perform latent profile analyses for all disorders individually, we were able to perform a subgroup analysis for the NF1 group. Finally, we included multiple instruments measuring ASD symptoms in our study. This allowed us to make comparisons between inherently different assessments and include information obtained by both clinicians and parents.

Our study also has several limitations. First, we only used two indicator variables in our ADOS model, which might have been insufficient. Wurpts and Geiser (2014) showed that the quality of latent class analysis models in general increases with more indicators, better indicator quality, and larger sample sizes. These findings are likely also true for LPA models (Wurpts & Geiser, 2014). In addition, by only looking at ADOS CSSs, we lose information on which specific symptoms are present in individuals. We still opted for the ADOS CSSs, despite losing the detailed focus on specific symptoms due to their comparability across different age groups. Second, we identified profiles based on symptom severity and did not consider other factors that might inform a diagnosis of ASD. Clinicians never solely base their diagnosis on a single assessment and take many other relevant factors into account, such as a patient’s daily functioning and impairment, developmental level, environmental factors, and general behavior and emotional problems in different situations. By basing our profiles solely on ASD symptoms, we ignore other factors that might drive a classification in a clinical setting.

### Future Directions

To address the drawback of fitting LPA models with only two indicator variables for the ADOS analysis, future studies could analyze ADOS modules individually, which allows for the fitting of item-level latent class models. Latent class models are similar to LPA models, but can handle categorical indicator variables (Magidson & Vermunt, 2004). This would provide a more fine-grained profile definition of ASD symptoms within rare neurodevelopmental disorders. However, obtaining large sample sizes in rare neurodevelopmental disorders is highly challenging. See the supplementary material, which includes Courbier et al. (2019) and Sherman et al. (2017), for our views on data sharing. Additionally, collaborations using joint samples would allow studies to be adequately powered to study the effects of genotype, such as FXS mosaicism (Budimirovic et al., 2020) or deletion vs. non-deletion genotypes in AS (Yang et al., 2021) on profile assignment.

The ENCORE VOLG database was set-up to follow patients over time and future studies should focus on the longitudinal heterogeneity of ASD symptoms. For example, group-based trajectory approaches can be used to detect latent developmental patterns (Nagin, 1999). Several studies used latent trajectory modelling to identify developmental patterns in ASD symptoms (Georgiades et al., 2022; Gotham et al., 2012; Szatmari et al., 2015; Venker et al., 2014; Visser et al., 2017; Waizbard-Bartov et al., 2022). Most of these studies found cognitive development to be lower in the latent trajectories with worse outcome. We found the same relationship between cognitive development and profile severity, albeit using cross-sectional data. Thus, factors associated to longitudinal trajectories may help to predict patient outcome, which might guide person tailored intervention. To our knowledge, there are no latent trajectory modelling studies that have investigated ASD symptomatology in FXS, AS, TSC, or NF1. The sample size of the VOLG database is currently not large enough to conduct these analyses, but we hope to obtain a sufficient sample size in the future in order to test whether we can identify development trajectories of ASD symptoms in children with FXS, AS, TSC, or NF1 or if symptoms remain stable in these syndrome groups.

Future studies should consider multiple behavioral domains when subtyping populations at risk for ASD. In our study, we only looked at SA and RRB symptoms, but individuals with an ASD are known to have an increased risk for other psychological problems as well (Lai et al., 2019). For example, individuals with ASD often show abnormal sensory processing and elevated rates of cognitive difficulties, behavioral symptoms, and mood disorders (Kodak & Bergmann, 2020; Oakley et al., 2021; Yates & Le Couteur, 2016). Exploring multi-domain symptom profiles would be in line with the current shift in the field toward the network perspective of psychopathological symptoms, in which psychiatric disorders are not thought of as coming from one specific cause, but rather as reflecting a complex network of interdependent symptoms. Identifying multi-domain profiles would allow clinicians to use a holistic approach regarding the symptoms in the treatment of individuals. Indeed, for all genetic syndromes included in the current study, psychological problems in multiple domains are common (Duis et al., 2022; Northrup et al., 1993; Protic et al., 2022; Torres Nupan et al., 2017). As our sample size continues to grow, we anticipate obtaining enough data to carry out multi-domain LPA for these syndromes.

We aimed to identify cross-syndromic ASD symptom profiles in children of genetic syndrome groups with a shared biological pathway. Although we did find evidence that some syndrome groups are more severely affected than others, our results show that there is a substantial overlap in ASD symptoms overlap *between* syndrome groups but they also vary significantly *within* syndrome groups. This absence of syndrome-specific behavioral ASD phenotypes stresses the need a for more personalized diagnostic and treatment approaches for children with a rare genetic disorder.

Finally, to get a better understanding of the differences in ASD symptom manifestation between biological males and biological females (Loomes et al., 2017; Narzisi et al., 2018), future studies should stratify their sample based on biological sex.

## Conclusion

To conclude, we found distinct latent ASD symptom profiles in a heterogeneous sample of children diagnosed with FXS, AS, TSC, and NF1. This supports the idea that ASD manifests heterogeneously and that meaningful subgroups based on strengths and weaknesses can be made, even for children with rare genetic syndromes that share biological pathways. Our results, especially for the ADOS analysis, stress the importance of person-centered treatment plans for ASD symptoms within these genetic syndrome groups. By focusing treatment on group-specific weaknesses we can optimize health care and improve future outcomes was no patient burden or privacy concern.

## Electronic supplementary material

Below is the link to the electronic supplementary material.

Supplementary Material 1
