# Supplementary material for: Autism Spectrum Disorder Symptom Profiles in Fragile X Syndrome, Angelman Syndrome, Tuberous Sclerosis Complex and Neurofibromatosis Type 1
Source: J Autism Dev Disord. 2024 Oct 12;56(2):793–807. doi: 10.1007/s10803-024-06557-2 (PMC12864356; doi:10.1007/s10803-024-06557-2)
Supplement: Supplementary file 1 — Supplementary Material 1 [file 10803_2024_6557_MOESM1_ESM.docx]

Autism Spectrum Disorder Symptom Profiles in Fragile X Syndrome, Neurofibromatosis Type 1, Angelman Syndrome and Tuberous Sclerosis Complex.

Journal of Autism and Developmental Disorders

Kyra Lubbers^*^, Kamil R. Hiralal^*^, Gwendolyn C. Dieleman, Doesjka A. Hagenaar, Bram Dierckx, Jeroen S. Legerstee, Pieter F.A. de Nijs, André B. Rietman, Rianne Oostenbrink, Karen G.C.B. Bindels-de Heus, Marie-Claire Y. de Wit, Manon H.J. Hillegers, Leontine W. ten Hoopen, Sabine E. Mous

* These authors have contributed equally to this publication and share first authorship

Correspondence

Sabine E. Mous, PhD, ENCORE Expertise Center for Neurodevelopmental Disorders, Erasmus MC, Rotterdam, The Netherlands, Department of Child and Adolescent Psychiatry and Psychology, Erasmus MC, Rotterdam, The Netherlands. Child Brain Center, Erasmus MC, Rotterdam, The Netherlands, s.mous@erasmusmc.nl

*S*upplementary material

*Validation using k-means*

*ADOS*

The k-means clusters found, are slightly different from the LPA profiles. We found a non-spectrum cluster (*n* = 207), an SA cluster (*n* = 100), a moderate severity cluster (*n* = 136), and an ASD cluster (*n* = 94) (see Figure S1). Individuals in the non-spectrum cluster have scores in the ADOS non-spectrum range. Individuals in the SA cluster are affected in the SA domain at the ADOS autism severity level. Individuals in the moderate severity cluster have a mean SA score just above the ADOS spectrum cut-off and a mean RRB score in the ADOS autism range. Individuals in the ASD cluster have scores within the ADOS autism range on both domains. Figure S2 displays the distribution of individuals across the LPA and k-means analyses. There is variation in the profile and cluster classification between the two analysis methods. The children assigned to the RRB profile are assigned to the non-spectrum cluster (53.1%) and the SA cluster (46.9%). Children assigned to the SA profile are distributed among the non-spectrum cluster (52.5%), SA cluster (7.5%), and elevated severity cluster (40.0%). The children in the ASD profile are classified in the SA cluster (28.4%), elevated severity cluster (25.5%), and ASD cluster (46.1%). All the children in the non-spectrum profile are assigned to the non-spectrum cluster.

*SRS*

The fit indices showed that k = 4 is a reasonable number of clusters. A k-means clustering model with four clusters is highly similar to our LPA model (Figure S3). Again, we found a non-clinical symptom cluster (*n* = 151), a mild symptom cluster (*n* = 129), a moderate symptom cluster (*n* = 117), and a severe symptom cluster (*n* = 68). Individuals in the non-clinical symptom cluster have T-scores in the non-clinical range on all SRS domains. Individuals in the mild symptom cluster show mild symptoms in the social cognition and social communication domains, but no symptoms in the other domains. Individuals in the moderate symptom cluster show mild symptoms in the social motivation domain, moderate symptoms in the social awareness, social cognition, and social communication domains, and severe symptoms in the autistic mannerisms domain. Individuals in the severe symptom cluster show severe symptoms in all SRS domains. In addition, Figure S4 shows the distribution of the children across the LPA profiles and k-means clusters. The percentage of children that is classified in the same LPA profile as the corresponding k-means cluster ranges from 63.4% for the mild symptom profile to 100% for the severe symptom profile.

*Subgroup analyses*

We repeated the LPA once for the NF1 group alone, and once for the FXS, AS and TSC groups combined (see table S1). We then qualitatively compared the results from our main analyses with our subgroup analyses. After removal of outliers (ADOS: n = 3; SRS: n = 3), the NF1 subgroup consisted of 275 children (53% male, mean age (SD) = 7.1 years(3.5)) for the ADOS and 263 children (58% male, mean age (SD) = 7.0 years (3.4)) for the SRS. The subgroup with FXS, AS and TSC combined contained no outliers and consisted of ADOS data for 259 children (n FXS = 54, n TSC = 112, n AS = 93, 57% male, mean age (SD) = 9.0 years(5.0)) and SRS data for 205 children (n FXS = 50, n TSC = 100, n AS = 55, 56% male, mean age (SD) = 9.1 years(5.1)).

*ADOS*

Similar to our main analysis, the five-profile solution resulted in two profiles that could not be interpreted as clinically different from each other. Therefore, we chose the four-profile solution as our final model. A MANOVA showed that the profiles were significantly different in CSSs (F(6,542) = 115.40, p < 0.001 η^2^_p_ = 0.56). The profile plots are shown in Figure S5.A. The profiles are interpreted as a non-spectrum profile (n = 69, 40.8% male, mean IQ = 89.6, epilepsy rate = 1.4%), a spectrum SA profile (n =77, 58.4% male, mean IQ = 84.5, epilepsy rate = 5.2%), a spectrum SA and RRB profile (n = 79, 60.8% male, mean IQ = 83.9, epilepsy rate = 5.1%), and a spectrum SA and severe RRB profile (n = 50, 64.0% male, mean IQ = 84.0 epilepsy rate = 8%). Individuals in the non-spectrum profile have CSSs below the ASD thresholds for both ADOS domains. Individuals in the spectrum SA profile have an SA CSS in the ASD range and an RRB CSS in the non-spectrum range. The spectrum SA and RRB profile shows CSSs in the ASD range for both domains. The spectrum SA and severe RRB profile is characterized by an SA CSS in the ASD range and an RRB CSS in the autism range. Similar to our main analysis, the individuals with NF1 are roughly evenly distributed across the profiles with a spectrum SA and RRB profile (28.7%), a spectrum SA profile (28.0%), a non-spectrum profile (25.1%), and a spectrum SA and severe RRB profile (18.2%). All fit statistics supported a four-profile model as the best fit. A MANOVA showed that the profiles were significantly different on the ADOS CSSs, F(6,510) = 79.59, p < .001, η^2^_p_ = 0.48. The profiles (Figure S1.B) are interpreted as a spectrum RRB profile (n = 27, 44.4% male, mean IQ = 54.5), a spectrum SA profile (n = 57, 42.1% male, mean IQ = 66.8), a severe RRB profile (n = 32, 59.3% male, mean IQ = 39.5), and an ASD profile (n = 142, 64.3% male, mean IQ = 35.8). Individuals in the spectrum RRB profile show an RRB-CSS in the ASD range and an SA-CSS in the Non-spectrum range. Individuals in the spectrum SA profile show an SA-CSS in the ASD range and an RRB-CSS in the Non-spectrum range. The severe RRB profile is characterized by an RRB-CSS in the autism range and a SA-CSS in the non-spectrum range. The ASD profile shows CSSs in the autism range for both domains. Epilepsy rates range from 62.9% to 74.1% across profiles, with the lowest epilepsy rate belonging to the ASD profile and the highest epilepsy rate to the RRB profile. The majority of individuals with FXS are classified in the ASD profile (81.5%). The rest of the individuals with FXS are evenly distributed among the other profiles. The majority of individuals with AS are classified in the ASD profile (54.8%). The rest of the AS group is mainly distributed among the severe RRB and the spectrum SA profiles (21.5% and 15.1% respectively). The majority of the TSC group is distributed between the ASD and spectrum SA profiles (42.9% and 35.7% respectively).

*SRS*

We chose the four-profile solution as our final model, as four out of the five goodness-of-fit statistics favor this model. A MANOVA shows that the profiles differ significantly in SRS T-scores, F(15, 771) = 29.90, p < 0.001, η^2^_p_ = 0.37. The profiles (Figure S2.A) are interpreted as a low symptom profile (n = 42, 47.6% male, mean IQ = 87.7, epilepsy rate = 0%), a non-clinical symptom profile (n = 112, 57.1% male, mean IQ = 88.5, epilepsy rate = 1.8%.), a mild symptom profile (n = 69, 60.9% male, mean IQ = 86.0, epilepsy rate = 8.7%), and a moderate/severe symptom profile (n = 40, 47.5% male, mean IQ = 77.7, epilepsy rate = 10%). Individuals in both the low symptom profile and the non-clinical symptom profile have T-scores in the non-clinical range, but the non-clinical symptom profile scores are closer to the mild symptom threshold than those in the low symptom profile. Individuals in the mild symptom profile display T-scores in the normal range for the social awareness, social motivation, and autistic mannerisms, and mild symptoms in the social cognition and social communication. Individuals in the moderate/severe symptom profile show moderate symptoms in the social awareness and social motivation domains and severe symptoms in the social cognition, social communication, and autistic mannerisms domains. The majority of the individuals with NF1 fall in the subclinical profiles, with 41.6% in the non-clinical profile and 15.6% in the low symptom profile. The mild symptom profile makes up 25.6% of the sample and the smallest group falls within the moderate/severe symptom profile (14.9%).

We found a three-profile model as the best fit, supported by all indices except for the BIC. A MANOVA showed that the SRS T-scores differed significantly between profiles, F(10,398) = 33.66, p < .001, η^2^_p_ = 0.46. The profiles (Figure S2.B) are interpreted as follows: a subclinical profile (n = 43, 55.8% male, mean IQ = 69.9, epilepsy rate = 55.8%), a moderate symptom profile (n = 148, 59.5% male, mean IQ = 41.7, epilepsy rate = 68.9%), and a severe symptom profile (n = 14, 21.4% male, M_IQ_ = 50.9, epilepsy rate = 35.7%). With the exception of the autistic mannerisms T-score, which just reaches the mild symptom threshold, all SRS T-scores of individuals in the subclinical profile fall within the subclinical range. Individuals in the moderate and severe symptom profiles display T-scores in the moderate and severe range, respectively, on all subscales. Almost all individuals with AS are classified in the moderate symptom profile (94.5%). The majority of the individuals with FXS and TSC are also classified in the moderate symptom profile (68.0% and 62.0% respectively). The remaining individuals with FXS and TSC are mainly classified in the subclinical profile (22.0% and 30.0% respectively).

*Statement on data sharing*

The problem of small sample sizes in rare genetic disorders be overcome by (inter)national data sharing, which could lead to large joint sample sizes. However, sharing of health data has to adhere to international regulations and raises issues on anonymity and trust in third parties (Courbier et al., 2019). Nevertheless, sharing data will lead to increased power to find statistical effects within groups of patients with rare diseases, which is crucial for improving treatment of these diseases. Courbier et al. (2019) showed that rare disease patient groups are motivated to share their data, despite the privacy concerns. Given the benefits of increased sample sizes and willingness of the patient groups to share data, researchers should explore options on how to share their data (such as sharing summary statistics or sharing anonymized data) while adhering to international regulations on privacy. For example, parent-reported data could be stored in national clinical data registries, such as FORWARD for FXS (Sherman et al., 2017) and the TSC Natural History Database (TSC Alliance), and a harmonized syntax could then be used for meta-analysis. In this way, large joint sample sizes could be achieved with relative ease, while making it possible to account for cultural
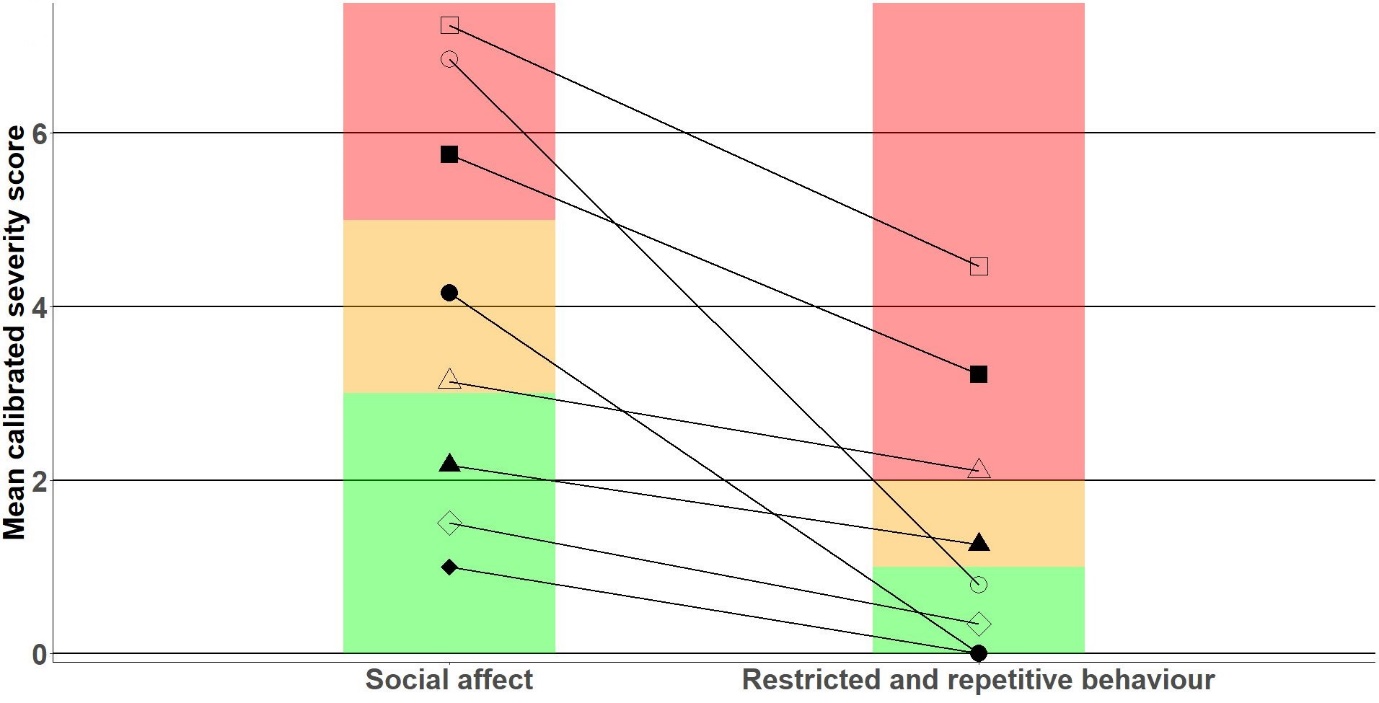
differences. These databases can also be used to replicate our findings.

**Fig S1.** a) Comparison of the LPA and k-means cluster analysis of the ADOS data. ■ = Higher severity profile, . ♦ = non-spectrum profile, ● = SA profile, ▲ = RRB profile, ■ = ASD profile, ◊ = non-spectrum cluster, ○ = SA cluster, Δ = moderate severity cluster, 🞏 = ASD cluster. Green indicates symptoms in the non-clinical range, orange in the ASD range, and red in the autism range (Lord, 2012)

**Fig S2.** Distributions of profile assignment across statistical method for the ADOS models.


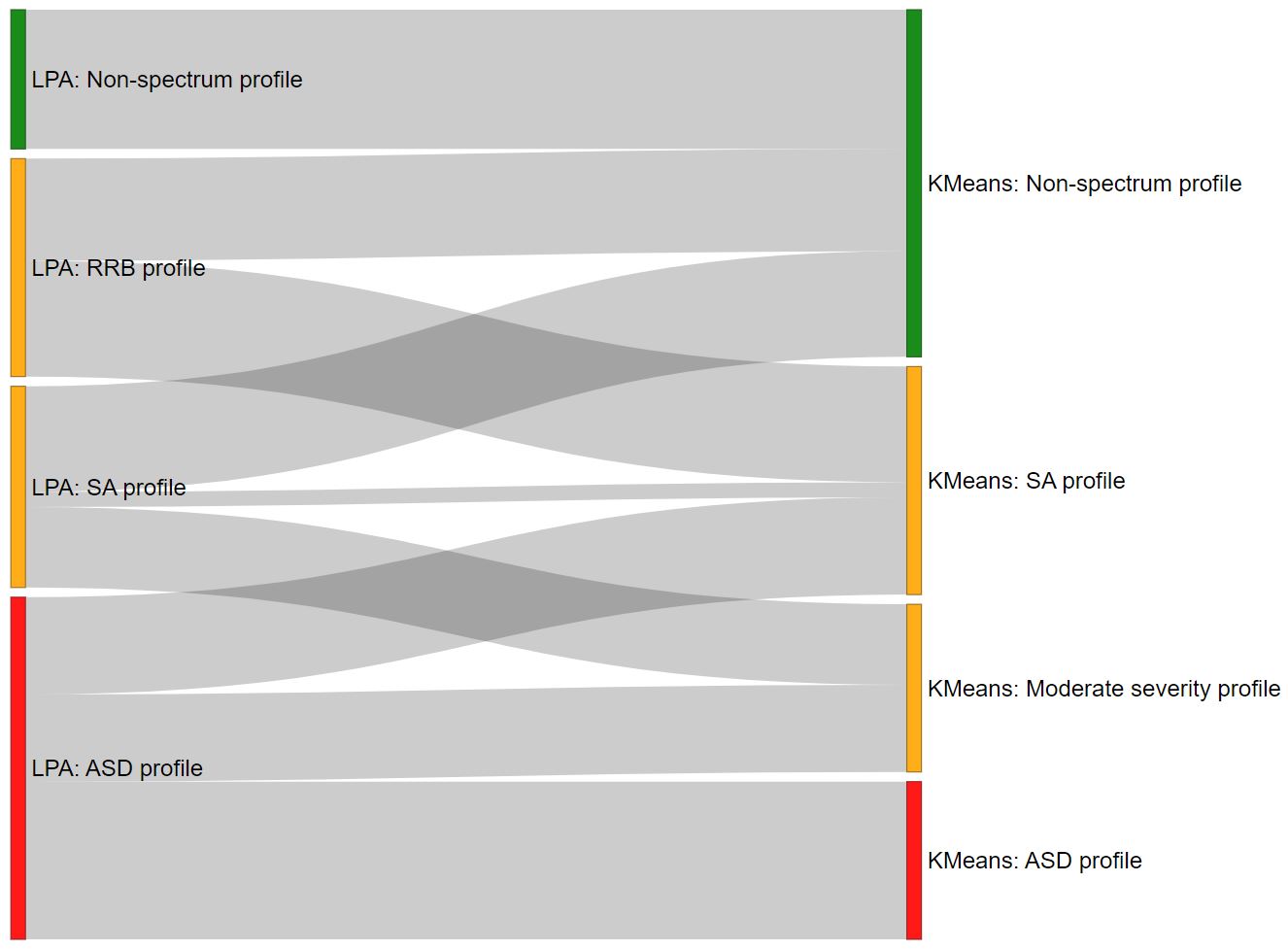


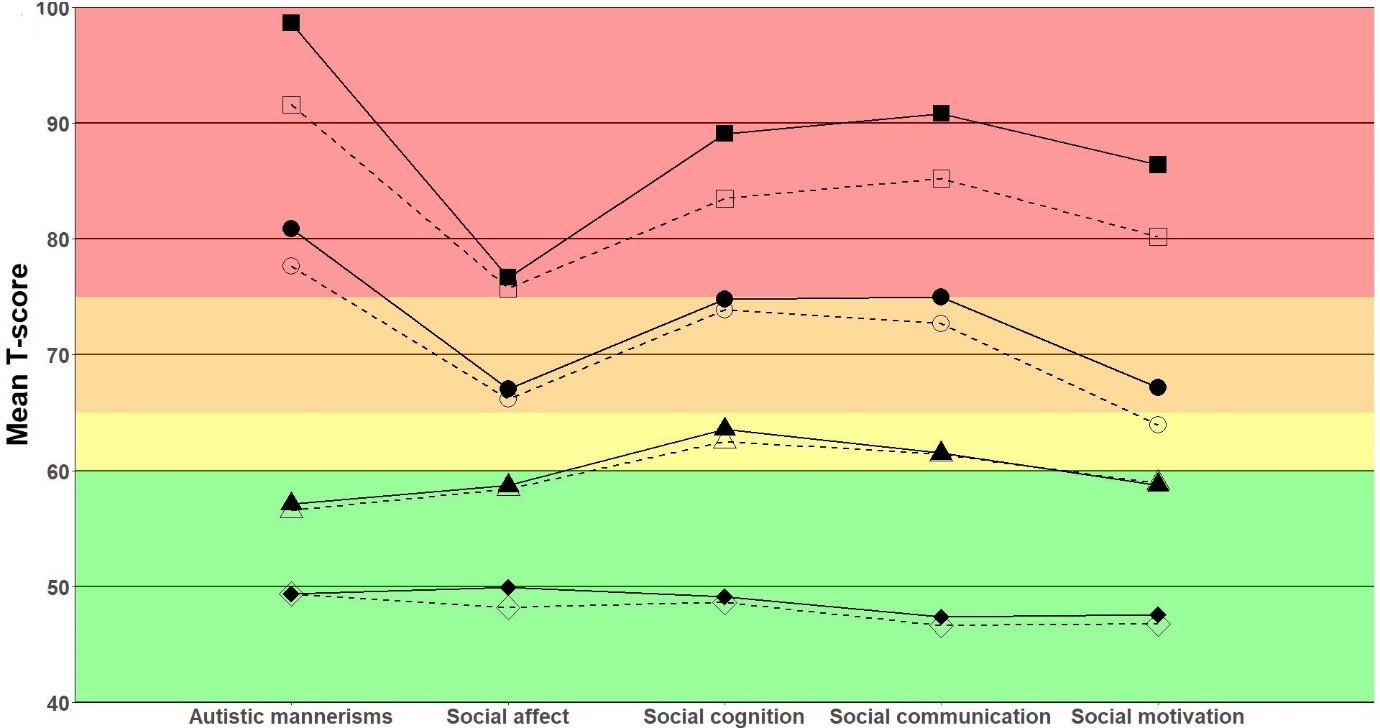

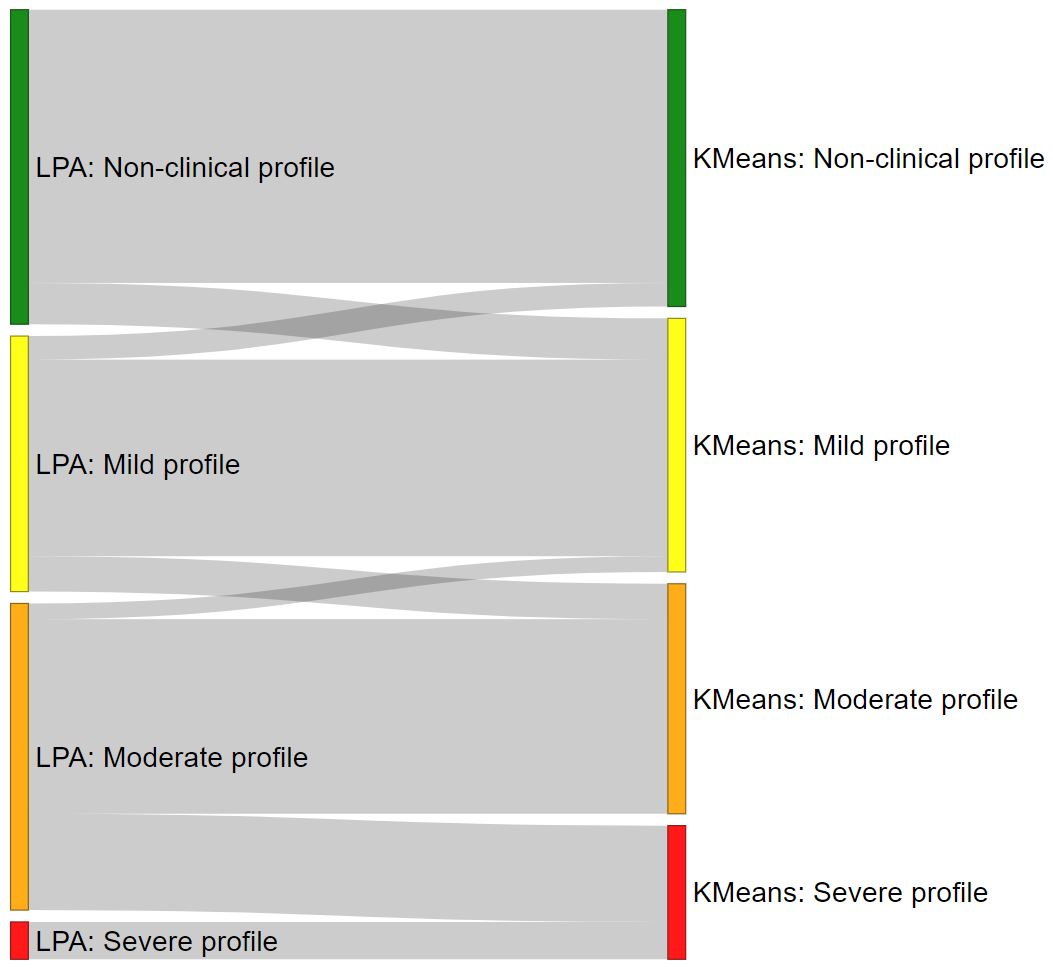


**Fig S4.** Distributions of profile assignment across statistical method for the SRS models.

**Figure S3.** Comparison of the LPA and K-means analysis of the SRS data. ■ = severe profile, ● = moderate profile, ▲ = mild profile, ♦ = non-clinical profile, 🞏 = severe cluster, ○ = moderate cluster, Δ = mild cluster, ◊ = non-clinical cluster. Green indicates symptoms in the non-clinical range, yellow in the mild range, orange in the moderate range, and red in the severe range (Bruni, 2014).


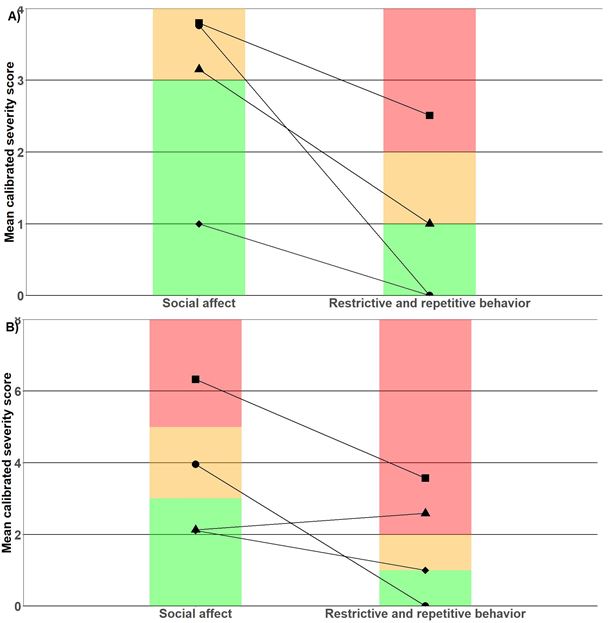


**Fig S5.** a) profile plots of the LPA ADOS model of the NF1 analysis. ■ = Higher severity profile, ▲ = spectrum profile, ● = SA profile, ♦ = non-spectrum profile. b) profile plots of the LPA ADOS model of the FXS, AS, and TSC groups combined. *■* = ASD profile, ▲ = High RRB profile, ● = SA profile, ♦ = Mild RRB profile. . Green indicates symptoms in the non-clinical range, orange in the ASD range, and red in the autism range (Lord, 2012)


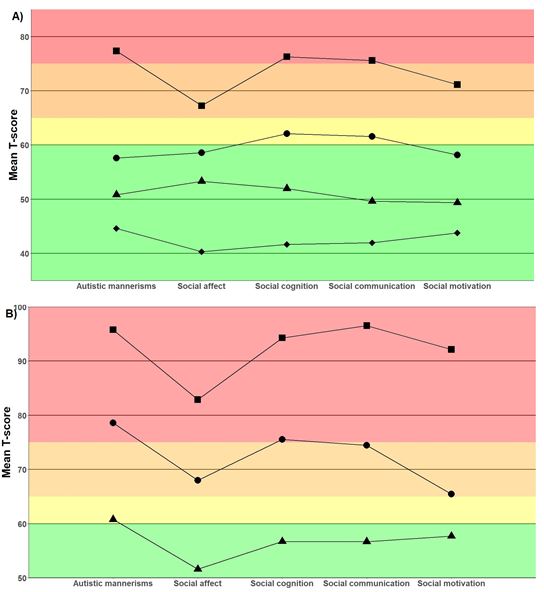


**Fig S6***.* a) profile plots of the LPA SRS model of the NF1 analysis. ■ = moderate/severe symptom profile, ● = mild symptom profile, ▲ = non-clinical profile, ♦ = low symptom profile. b) profile plots of the LPA SRS model of the FXS, AS, and TSC groups combined. B) ■ = Severe symptom profile, ● = moderate symptom profile, ▲ = Sub-clinical symptom profile. Green indicates symptoms in the non-clinical range, yellow in the mild range, orange in the moderate range, and red in the severe range (Bruni, 2014).

| **Table S1** | | | | | | | |  |
| --- | --- | --- | --- | --- | --- | --- | --- | --- |
| *LPA fit statistics for the subgroup analyses* | | | | | | | |  |
|  |  | **BIC** | **SABIC** | **AIC** | **Log likelihood** | **Entropy** | **LRT-statistic** | |
| *ADOS NF1* | |  |  |  |  |  |  | |
|  | 1-profile | 2026.87 | 2011.02 | 2008.79 | -999.40 | 1.00 | - | |
|  | 2-profiles | 1570.82 | 1535.94 | 1531.04 | -754.52 | 0.99 | 489.75* | |
|  | 3-profiles | 1437.35 | 1383.45 | 1375.87 | -670.93 | 0.84 | 167.17* | |
|  | 4-profiles | 865.32 | 792.39 | 782.13 | -368.07 | 0.97 | 605.74* | |
|  | 5-profiles | 803.86 | 711.91 | 698.98 | -320.48 | 0.97 | 95.15* | |
| *ADOS FXS, TSC, and AS* | | |  |  |  |  |  | |
|  | 1-profile | 2286.16 | 2270.31 | 2268.37 | -1129.19 | 1.00 | - | |
|  | 2-profiles | 2247.77 | 2212.90 | 2208.65 | -1093.32 | 0.82 | 71.73* | |
|  | 3-profiles | 2151.84 | 2097.94 | 2091.37 | -1028.69 | 0.90 | 129.27* | |
|  | 4-profiles | 2123.80 | 2050.88 | 2042.00 | -998.00 | 0.92 | 61.38* | |
|  | 5-profiles | 2246.78 | 2154.84 | 2143.63 | -1042.82 | 0.84 | -89.64 | |
| *SRS NF1* | |  |  |  |  |  |  | |
|  | 1-profile | 9368.00 | 9304.59 | 9296.55 | -4682.28 | 1.00 | - | |
|  | 2-profiles | 9272.14 | 9186.53 | 9175.69 | -4560.84 | 0.73 | 134.87* | |
|  | 3-profiles | 9288.33 | 9180.53 | 9166.88 | -4549.44 | 0.67 | 22.81* | |
|  | 4-profiles | 9294.66 | 9164.67 | 9148.20 | -4533.10 | 0.76 | 32.67* | |
|  | 5-profiles | 9319.37 | 9167.75 | 9147.90 | -4525.95 | 0.73 | 14.30* | |
| *SRS FXS, TSC, and AS* | | |  |  |  |  |  | |
|  | 1-profile | 7763.04 | 7699.68 | 7696.58 | -3828.29 | 1.00 | - | |
|  | 2-profiles | 7780.93 | 7695.39 | 7691.21 | -3818.61 | 0.76 | 19.37 | |
|  | 3-profiles | 7789.48 | 7681.76 | 7676.50 | -3804.25 | 0.87 | 28.71* | |
|  | 4-profiles | 7822.74 | 7692.84 | 7686.50 | -3802.25 | 0.64 | 4.00 | |
|  | 5-profiles | 7821.61 | 7669.53 | 7662.11 | -3783.05 | 0.83 | 38.39* | |
| *Note.* Lower absolute BIC, SABIC, AIC, and log likelihood values indicate better fit. * = bootstrap *p*-value < .05. | | | | | | | | |

| **Table S2** | | | | | | |
| --- | --- | --- | --- | --- | --- | --- |
| *Multinomial logistic regression results for the ADOS profiles* | | | | | | |
|  |  | *B* | SD | Wald | OR | 95% CI |
| *ASD Profile* | | | | | | |
|  | Age | 0.01 | 0.04 | 0.14 | 1.00 | [0.93-1.09] |
|  | IQ/DQ | -0.5 | 0.01 | -5.31 | 0.61 | [0.51-0.73] |
|  | Female | -1.35 | 0.32 | -4.20 | 0.26 | [0.14-0.49] |
|  | Epilepsy | 0.06 | 0.56 | 0.10 | 3.15 | [0.35-3.15] |
|  | Group (NF1 reference) |  |  |  |  |  |
|  | FXS | 15.49 | 0.31 | 49.87 | 5.33*10^6^ | - |
|  | AS | 0.48 | 0.81 | 0.59 | 1.61 | [0.33-7.89] |
|  | TSC | 1.24 | 0.56 | 2.19 | 3.46 | [1.44-10.49] |
| *RRB Profile* | | | | | | |
|  | Age | 0.00 | 0.04 | -0.03 | 0.99 | [0.93-1.08] |
|  | IQ/DQ | -0.1 | 0.01 | -1.68 | 0.87 | [0.73-1.02] |
|  | Female | -0.60 | 0.29 | -2.05 | 0.55 | [0.31-0.97] |
|  | Epilepsy | 0.26 | 0.54 | 0.47 | 1.29 | [0.45-3.72] |
|  | Group (NF1 reference) |  |  |  |  |  |
|  | FXS | 13.49 | 0.38 | 35.01 | 7.21*10^5^ | - |
|  | AS | 0.24 | 0.81 | 0.30 | 1.27 | [0.26-6.20] |
|  | TSC | 0.30 | 0.55 | 0.54 | 1.36 | [0.46-3.92] |
| *SA Profile* | | | | | | |
|  | Age | -0.01 | 0.04 | -0.30 | 0.99 | [0.92-1.07] |
|  | IQ/DQ | -0.1 | 0.01 | -1.10 | 0.91 | [0.77-1.08] |
|  | Female | -0.67 | 0.30 | -2.25 | 0.51 | [0.29-0.92] |
|  | Epilepsy | 0.13 | 0.54 | 0.24 | 1.14 | [0.39-3.30] |
|  | Group (NF1 reference) |  |  |  |  |  |
|  | FXS | 13.24 | 0.44 | 30.10 | 5.63*10^5^ | - |
|  | AS | 0.24 | 0.83 | 0.30 | 1.36 | [0.27-6.89] |
|  | TSC | 0.91 | 0.53 | 1.72 | 2.50 | [4.68-604] |
| *Note.* Some confidence intervals are not provided here, since they are too large to fit the table. IQ/DQ = Intelligence or developmental quotient (units of 10), FXS = Fragile X Syndrome, AS = Angelman Syndrome, TSC = Tuberous Sclerosis Complex, NF1 = Neurofibromatosis Type 1 | | | | | | |

| **Table S3** | | | | | | |
| --- | --- | --- | --- | --- | --- | --- |
| *Multinomial logistic regression results for the SRS profiles* | | | | | | |
|  |  | *B* | SD | Wald | OR | 95% CI |
| *Severe symptom Profile* | | | | | | |
|  | Age | 0.16 | 0.06 | 2.56 | 1.17 | [1.04-1.32] |
|  | IQ/DQ | -0.55 | 0.01 | -3.79 | 0.58 | [0.43-0.77] |
|  | Female | 2.06 | 0.72 | -2.88 | 7.86 | [1.93-32.0] |
|  | Epilepsy | -0.55 | 0.87 | -0.63 | 0.57 | [0.1-3.18] |
|  | Group (NF1 reference) |  |  |  |  |  |
|  | FXS | 5.58 | 1.34 | 4.16 | 266.3 | [19.2-3704] |
|  | AS | 14.75 | 1.09 | 13.56 | 2.55 * 10^5^ | - |
|  | TSC | 3.97 | 1.24 | 3.21 | 53.2 | [4.68-604] |
| *Moderate symptom Profile* | | | | | | |
|  | Age | 0.07 | 0.04 | 1.85 | 1.07 | [1.00-1.16] |
|  | IQ/DQ | -0.46 | 0.01 | -5.48 | 0.63 | [0.53-0.74] |
|  | Female | -0.03 | 0.30 | -0.10 | 0.97 | [0.54-1.75] |
|  | Epilepsy | 0.74 | 0.53 | 1.41 | 2.10 | [0.75-5.90] |
|  | Group (NF1 reference) |  |  |  |  |  |
|  | FXS | 2.76 | 0.80 | 3.45 | 15.80 | [3.29-75.38] |
|  | AS | 14.30 | 0.61 | 23.42 | 1.62 * 10^6^ | - |
|  | TSC | 1.38 | 0.53 | 4.61 | 3.99 | [1.412.73-114.2811] |
| *Mild symptom Profile* | | | | | | |
|  | Age | 0.07 | 0.04 | 2.03 | 1.07 | [1.00-1.16] |
|  | IQ/DQ | -0.16 | 0.01 | -2.10 | 0.85 | [0.74-0.99] |
|  | Female | -0.33 | 0.26 | -1.29 | 0.72 | [0.43-1.19] |
|  | Epilepsy | 0.41 | 0.50 | 0.82 | 1.50 | [0.57-3.97] |
|  | Group (NF1 reference) |  |  |  |  |  |
|  | FXS | 2.11 | 0.80 | 2.62 | 39.80 | [1.70-39.80] |
|  | AS | 14.32 | 0.65 | 22.15 | 5.94 * 10^6^ | - |
|  | TSC | 1.33 | 0.47 | 2.79 | 3.79 | [2.241.49-10.29.64] |
| *Note.* Some confidence intervals are not provided here, since they are too large to fit the table. IQ/DQ = Intelligence or developmental quotient (units of 10), FXS = Fragile X Syndrome, AS = Angelman Syndrome, TSC = Tuberous Sclerosis Complex, , NF1 = Neurofibromatosis Type 1 | | | | | | |
